# Annotations

**Published:** 1889-05-11

**Authors:** 


### ANNOTATIONS.

**Medical Authority.**

The doctor is a much consulted man, inevitably. In the minds of his patients he occupies the judicial bench all day long and every day. The position is deeply responsible, and not without its drawbacks. It is not every doctor who is blessed with a judicial mind; and even the most judicial mind in the world is not always in a judicial mood. The physician's errors of judgment—and what physician does not make them?—may be very costly to the patient in a variety of ways. An expensive line of treatment may be ordered when a simpler would have sufficed; and the obedience of the patient and his friends may cause serious pressure at home. Or the failure of the physician to grasp the immediate urgency of the case, and to insist with unyielding firmness upon the prompt carrying out of the advice given, may be the one broken link in a chain of sequences which is the cause of the patient's death. All work should be well done—done with the whole soul; but emphatically and constantly such work as that of the doctor. Was it Richard Steele who said of his wife that "to have known her well was a liberal education"? It is certainly true that to have practised medicine honestly and intelligently for a dozen years is one of the best schools of discipline through which any man can have passed. The doctor must be judicial, or at least appear to be so. His final and decisive authority is appealed to twenty times a day. Is it to be wondered at that, being human, he sometimes assumes a too dogmatic tone, and is a little impatient of contradiction? But the present age is not favourable to the claims of individual infallibility. Princes, popes, prime ministers, and popular preachers are all passed through the critical mill, and we must not take it amiss if physicians, too, are ground small by the spirit of the times. There is still, however, quite enough of authority conceded to the doctor to satisfy any but the most exacting mind, too much, indeed, for comfort if his practice be large. The great point is for the medical man to recognise and steadfastly bear in mind the weight which is attached even to his lightest words, and to constantly practise the habit of speaking as if all his utterances were listened to, not only by his patients, but by the Censors Board of the College of Physicians.

**Vaccination on its Trial.**

It has been decided to hold a commission of enquiry which shall embrace the whole scope of the compulsory vaccination question. With this decision no class of persons can feel more satisfaction than medical men. If any faith is firm in these days of general doubt, it is that of doctors in the doctrine and practice of Jennerism. There should be no insuperable difficulty in the obtaining of sufficient data on which to found reliable conclusions. The past may be compared with the present, and the present with itself. If a scientific commission and the evidence of experts be of any value at all, a verdict should be arrived at which will settle the question for a very long time. There is a kind of incipient tendency to schism among some thoughtful members of the medical profession. Those gentlemen will now have an opportunity, which no doubt they will gladly embrace, of setting forth the grounds of their dissent, and of making converts, or of being themselves converted. On so broad and important a practical question as that of universal compulsory vaccination the medical profession itself should at least speak with no uncertain sound. Individual doctors should understand that a grave responsibility rests upon them, and should by no means enter upon a course which will disquiet the public mind without good reason. We are altogether in favour of critical research and unfettered enquiry into every conceivable branch of human knowledge and practice. Nothing that we know of is so fixed and certain as that it may not be submitted to investigation and criticism. But a man who, without solid grounds to go upon, rashly raises the flag of revolution in any department of human activity is a flippant, and may be a dangerous fool. "Light, more light," let us have by all means, from whatever quarter it may come. But it must be real and external light, and not merely the subjective sensations which may affect a man's own particular optic nerves. The vaccination question is eminently one of hard, concrete fact. Vaccination does or does not diminish the liability to smallpox; it does or does not modify the effect of the smallpox poison in the system; it is or it is not a means of partial or of complete protection against the ravages of a most painful, repulsive, and dangerous disease. If all, or nearly all its advocates claim for it be true, then its universal application is at least eminently desirable, and probably even compulsion itself is a justifiable measure. If, on the other hand, the opponents prove their case, and show that, on the whole, compulsory vaccination does more harm than good, or at least that its asserted value is wholly incapable of proof, then compulsion must go. It will, of course, still be open for medical men and other believers to prove their faith by their works, and to have themselves and their own children vaccinated. We venture to prophesy that if the anti-vaccinationists have their way, not one medical man in a thousand will neglect the thorough vaccination of his own children, or the revaccination of himself in epidemic times.

**The Moral Effects of Anæsthetics.**

From Japan comes a story that sets one thinking. An old lady, 60 years of age, was attacked by a disease which was curable only by a severe surgical operation, which she professed herself willing to undergo. When the operation was to take place, the operator's assistant prepared to administer chloroform. The old lady asked what was the use of this medicine, and on learning that its function was only to deaden pain, she resolutely refused to have anything to do with it. To evade pain was, in her estimation, despicable and cowardly, unworthy of the old Japanese spirit, which enabled a man to compose a poem in the agonies of the "happy despatch." She would undergo the operation, but without chloroform. So she lay down on the operating table, and had four deep-seated abscesses removed and twenty stitches put in, without making a movement or uttering a groan. When all was over, and the doctor asked her if she had suffered much pain, she opened her eyes and answered quietly that the cutting of live flesh was never without suffering. That was her only comment on what she had undergone, and she wished there and then to write letters to reassure her anxious friends. From the point of view of narrow common-sense, this old lady was very foolish to insist on enduring pain she could have avoided; but the idea on which her refusal to take the anæsthetic was based—that to evade pain that comes for good reason is cowardly—has something noble about it. Perhaps we who have ether every time a tooth is extracted err on the other side. We are so used to evading pain that we look on it, not merely as a misfortune, but as an injustice; and the habit of thus regarding physical suffering may affect our views on mental distress as well, and teach us that it is legitimate to get out of every sort of trouble. Mrs. Lynn Linton has lately argued that our indulgence in anæsthetics had an enervating effect, and not without at least a show of reason. The idea of shirking pain of any kind is apt to weaken the moral fibre. Without setting up the ascetic ideal, or meeting trouble half way, it is sometimes necessary to accept the fact that pain is a thing to be undergone without an anodyne, and to teach ourselves to bear it as quietly as may be.

